# To Our Advertising Agents

**Published:** 1876-12

**Authors:** 


*To our Advertising Agents.*—It is believed that THE SOUTHERN MEDICAL RECORD has the largest circulation of any medical journal published in the South, and it is read by all classes of the profession, not only in the South, but in all parts of the Union. It is therefore a splendid advertising medium for any and every kind of business that will interest doctors and druggists.

To advertising agents who receive our journal, we will state that they must procure business for THE RECORD if they would have it continued to their address the ensuing year.

Any who may desire to solicit business for our journal will please notify us of the same.

